# Comparison of excess deaths and laboratory-confirmed COVID-19 deaths during a large Omicron epidemic in 2022 in Hong Kong

**DOI:** 10.7189/jogh.15.04105

**Published:** 2025-04-11

**Authors:** Hualei Xin, Alexandra H T Law, Justin K Cheung, Yun Lin, Peng Wu, Zhongjie Li, Benjamin J Cowling, Weizhong Yang, Jessica Y Wong

**Affiliations:** 1 School of Population Medicine and Public Health, Chinese Academy of Medical Sciences & Peking Union Medical College, Beijing, China; 2 World Health Organization Collaborating Centre for Infectious Disease Epidemiology and Control, School of Public Health, Li Ka Shing Faculty of Medicine, The University of Hong Kong, Hong Kong Special Administrative Region, China; 3 Laboratory of Data Discovery for Health Limited, Hong Kong Science and Technology Park, New Territories, Hong Kong Special Administrative Region, China

## Abstract

**Background:**

Using an elimination strategy, Hong Kong was able to minimise COVID-19 mortality in 2020 and 2021, but a large epidemic caused by the Omicron variant occurred in 2022. We aimed to estimate the overall, age-, sex-, epidemic period- and cause-specific excess mortality in 2022 in Hong Kong and compare excess mortality to laboratory-confirmed COVID-19 mortality.

**Methods:**

We used negative binomial regression analysis to model time series of weekly all-cause and cause-specific deaths from 2010 to 2021 to predict the weekly number of deaths in 2022 against counterfactual baselines projected from the trends in the absence of a pandemic. The estimated excess deaths were compared with laboratory-confirmed COVID-19 deaths overall and by age and epidemic period.

**Results:**

We estimated that there were 13 500 (95% confidence interval (CI) = 13 400, 13 600) excess deaths in 2022, which was slightly higher than the 12 228 deaths recorded with laboratory-confirmed COVID-19, with the majority of the excess deaths and laboratory-confirmed deaths occurring among older adults. The increased number of excess deaths over laboratory-confirmed COVID-19 deaths was most substantial from February to April 2022 (a difference of 847 deaths), when the most prominent Omicron wave peaked. Most of the excess deaths (78%) were from respiratory causes, while 10% were from cardiovascular causes. A slight reduction in malignant neoplasm mortality was identified among older adults in 2022.

**Conclusions:**

A substantial increase in population mortality was identified in 2022 in Hong Kong, slightly larger than the laboratory-confirmed COVID-19 deaths. Deaths from COVID-19 may have displaced some deaths that would otherwise have occurred due to other causes.

Since the emergence of SARS-CoV-2 at the end of 2019, millions of COVID-19 deaths with infection of the virus have been recorded globally [1]. However, the reported number may not represent the mortality impact associated with the COVID-19 pandemic for several reasons. First, some COVID-19 deaths may be assigned to other causes due to misclassification or misdiagnosis, particularly during the early phase of the pandemic when the diagnostic capacity was more limited [2,3]. Second, there may have been indirect health impact due to the disruption of essential medical services due to pressure on the health-system [3], changes in medical behaviour resulting from difficulty in accessing health care services [4,5], or fear of getting infected in medical institutions or delays in seeking health care under stay-at-home policies or other social distancing measures [5,6]. Excess mortality is the difference between the observed and expected mortality obtained under conditions with no crisis [7,8]. This indicator has long been used to estimate the disease burden of past health crises or pandemics based on mortality time-series [8–10]. For the COVID-19 pandemic, the World Health Organization (WHO) estimated 14.83 million excess deaths in 2020 and 2021, which is 2.74 times higher than the reported deaths (5.42 million) from laboratory-confirmed SARS-CoV-2 infections [3].

Hong Kong adopted an elimination strategy during the COVID-19 pandemic in 2020 and 2021 [11]. The elimination strategy includes maximum action to exclude disease and eliminate community transmission. In contrast, the mitigation strategy focused on protecting vulnerable and high-risk groups while allowing transmission among low-risk groups, aims to avoid overwhelming health systems by flattening the epidemic curve or achieving herd immunity in the population [11]. In Hong Kong, individual and population interventions were introduced in 2020 and 2021, such as universal isolation of confirmed cases, strict quarantine of close contacts, school closures and other compulsory social distancing measures [12]. These measures effectively controlled pre-Omicron transmission resulting in 181 (2 per 100 000) local laboratory-confirmed COVID-19 deaths in 2020 and 30 (0.4 per 100 000) in 2021 [12]. However, our previous study showed an estimated 1800 excess deaths from diseases other than COVID-19 in 2020, potentially related to delays or reductions in health care seeking as a consequence of strict public health and social measures [5]. In contrast, New Zealand, where a similar elimination strategy as Hong Kong was implemented, observed a lower overall mortality rate than expected in 2020 [13]. For some countries in Europe and North America, a mitigation or suppression strategy for the COVID-19 pandemic was implemented initially [11]. For most of those countries, a higher number of excess deaths were identified, and the number was more than 30% higher than reported COVID-19 deaths [13].

In early 2022 Hong Kong experienced a very large Omicron epidemic in the early period of the year, with over two million confirmed COVID-19 cases (by RT-PCR or rapid antigen tests) and 10 000 deaths reported (136 per 100 000) [12]. We hypothesise that excess mortality would be identified during the large Omicron wave of the COVID-19 pandemic in 2022 and largely attributed to infection with COVID-19. In this study, we aimed to investigate mortality impact of COVID-19 over a much larger epidemic caused by Omicron variants in Hong Kong. We estimated the excess mortality in 2022 by age, sex, epidemic period, and cause of death, and comparing the excess mortality to the reported fatal cases with confirmed COVID-19.

## METHODS

### Sources of data

Individual death data from 2010 to 2022 were obtained from the Census and Statistics Department of Hong Kong. Weekly mortality data were aggregated into groups of age (0–4, 5–14, 15–44, 45–64, 65–79 and ≥80), sex, and cause of death. Cause of deaths were coded by the International Classification of Diseases Tenth Revision (ICD-10) and grouped into: all causes (A00-Z99), and the following major causes: respiratory diseases (J00-J99), malignant neoplasms (C00-C97), cardiovascular diseases (I00-I99), kidney diseases (N00-N07, N17-N19, N25-N27), external causes (S00-T88), diabetes mellitus (E10-E14), and other causes (including diseases of the digestive system, mental and behavioural disorders, certain infectious and parasitic diseases, diseases of the nervous system, *etc*.). Data on individual patients with laboratory-confirmation of SARS-CoV-2 in 2022 were obtained from the Hong Kong Hospital Authority (HA), Centre for Health Protection and Deaths Registry [14]. Epidemiological information of individual patients was provided in the database, including age, sex, date of laboratory confirmation, occurrence of death in-hospital or out-of-hospital. In this analysis, a laboratory-confirmed COVID-19 death was defined as a person who had a positive RT-qPCR result testing for SARS-CoV-2 with a respiratory specimen collected within 28 days before or seven days after the date of death. Excess mortality is the difference between the observed and expected mortality obtained under conditions with no crisis. To find the association between excess mortality for diseases other than COVID-19 and health care utilisation (represent indirect impact of the pandemic), weekly aggregated data on hospital admissions between 2010 and 2022 were collected from HA by age, sex, and cause of admission. The annual mid-year population data by age and sex between 2010 and 2022 were collected from the Census and Statistics Department [15].

### Statistical analysis

Weekly mortality risk was calculated by dividing the number of weekly deaths by mid-year populations in Hong Kong, by age, sex, and cause of death. Due to the long-term trend and seasonality shown from the historical data, we selected regression models accounting for temporal trend and seasonality rather than simply comparing the mortality in the study period with the mortality in the previous year. We applied negative binomial regression models to the time series of weekly number of all-cause and disease-specific deaths from 2010 to 2021, assuming the temporal mortality in 2010–21 extending to 2022. A group of models were developed using different Fourier terms (varying from 1 to 30) with or without the variable ‘week’ to explore the impact of seasonality and using calendar year and the week sequence (from the first week of 2010 to the last week of 2021) to indicate the temporal trends. The model’s performance was examined using the Akaike Information Criterion (AIC). In the regression model selected for the final analysis, we included the population size as an offset and accounted for long-term trends using the calendar year. Fourier terms of eight harmonics within one year with the variable ‘week’ were included in the regression models to allow for annual seasonality. Another covariate was included in the model to account for changes in the mortality trend in Hong Kong since the emergence of SARS-CoV-2 in January 2020. The COVID-19-associated excess mortality risk was calculated by subtracting the predicted weekly mortality risk from the fitted regression model from the observed weekly mortality risk.

In 2022, Hong Kong experienced two major epidemics driven by Omicron variants, including the largest wave during February – April and a smaller wave from November onwards. We defined weeks 1–26 as period 1 (wave 5), weeks 27–45 as period 2 (wave 6a), and weeks 46–52 as period 3 (wave 6b) [12]. The all-cause excess mortality was estimated for these three periods using the same method described above and compared with the reported COVID-19 mortality for each period.

To quantify the impact of health care utilisation on excess mortality of causes other than COVID-19 during the pandemic, an interrupted time-series analysis was used to estimate the weekly cause-specific hospitalisation reduction risk in 2022 based on historical data from 2010 to 2021, controlling seasonality, temporal trend and potential changing trend of hospitalisation after the occurrence of the COVID-19 pandemic. Pearson correlation coefficients (denoted by r) were estimated to indicate the relationship between weekly excess mortality and hospitalisation reduction risk in 2022 for causes including malignant neoplasms, cardiovascular diseases, kidney diseases, and diabetes mellitus. The analysis was also conducted for subcategories of respiratory and cardiovascular diseases, including pneumonia and influenza (J09-J18), chronic lower respiratory disease (J40-J47), heart disease (I00-I09, I11, I13, I20-I51), and cerebrovascular diseases (I60-I69).

Further technical details of the statistical methods are provided in the **Online Supplementary Document**. All statistical analyses were conducted in *R*, version 4.1.0 (R Foundation for Statistical Computing, Vienna, Austria).

## RESULTS

**Figure 1:**
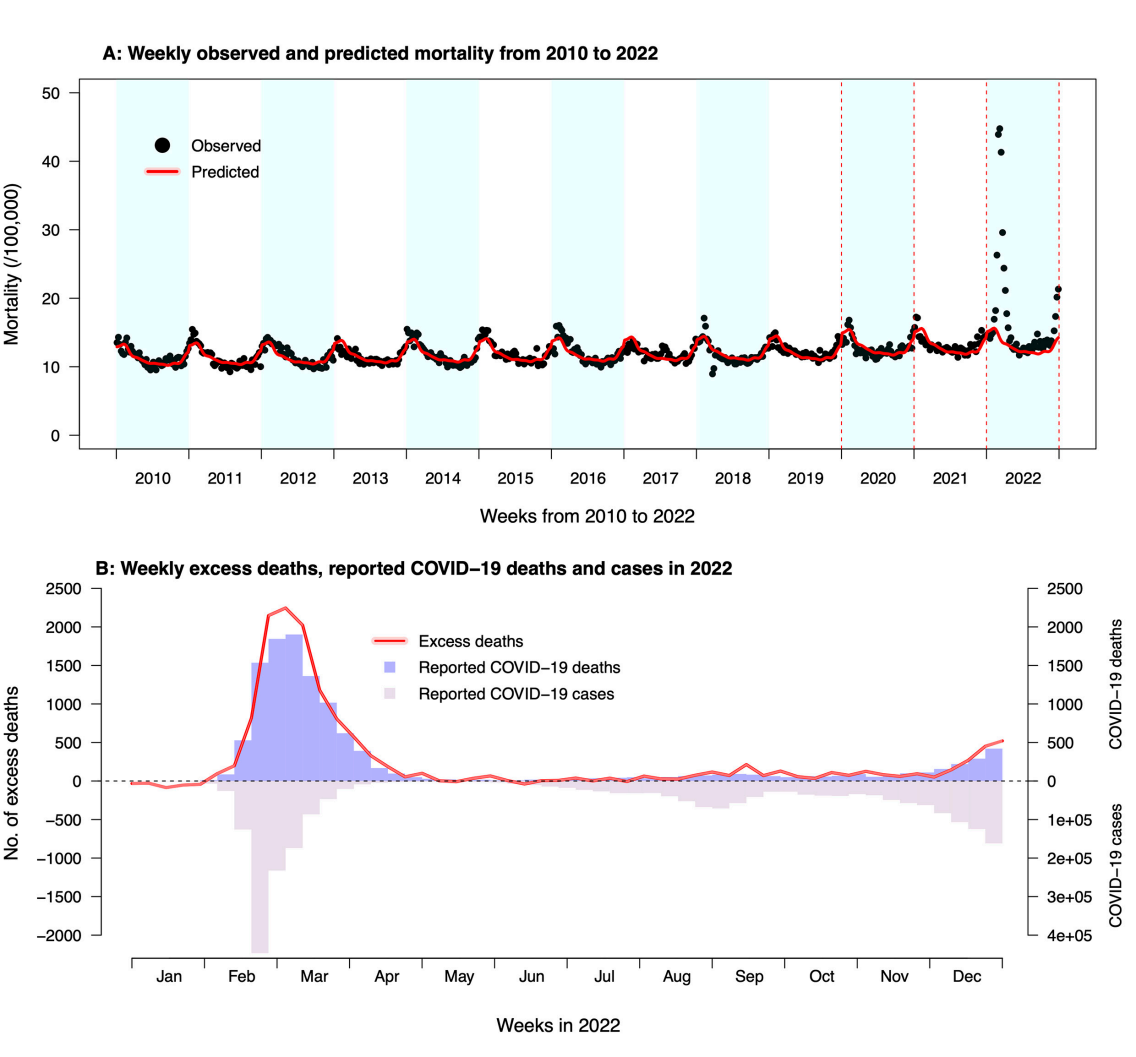
The overall trend of mortality and excess deaths in 2022 in Hong Kong. **Panel A.** Weekly observed (black dots) and predicted (red solid line and the pink shaded area show the mean predicted weekly mortality and 95% CIs, respectively) mortality from 2010 to 2022 in Hong Kong. **Panel B.** Predicted weekly numbers of excess deaths (red solid line and the pink shaded area show the mean weekly numbers of excess deaths and 95% CI, respectively), weekly numbers of reported COVID-19 deaths (purple bars) and weekly numbers of locally infected COVID-19 cases in Hong Kong in 2022. CI – confidence interval.

An overall increasing trend of annual mortality in Hong Kong was observed from 2010 through to 2022, with a slight increase from 596 per 100 000 population in 2010 to 645 in 2019, and a sharp rise to 864 per 100 000 population in 2022 (**Figure 1**, Panel A). Prior to 2022, the weekly mortality peaked in late December to January every year while a prominent peak was noted in February – March in 2022, in line with the trend in confirmed COVID-19 case numbers (**Figure 1**, Panels A–B). In 2022, people aged 80 years or above had the highest mortality, followed by individuals aged 65–79 years, and the mortality risk was generally higher in males than females. The top three causes of death in 2022 were respiratory diseases (302 per 100 000 population), malignant neoplasms (202 per 100 000 population), and cardiovascular diseases (152 per 100 000 population), which differed from 2020 and 2021 when malignant neoplasms (197 and 203 per 100 000 population in 2020 and 2021, respectively) were the leading cause, followed by respiratory (151 and 157 per 100 000 population) and cardiovascular (137 and 140 per 100 000 population) diseases (Table S1 in the **Online Supplementary Document**).

**Figure 2:**
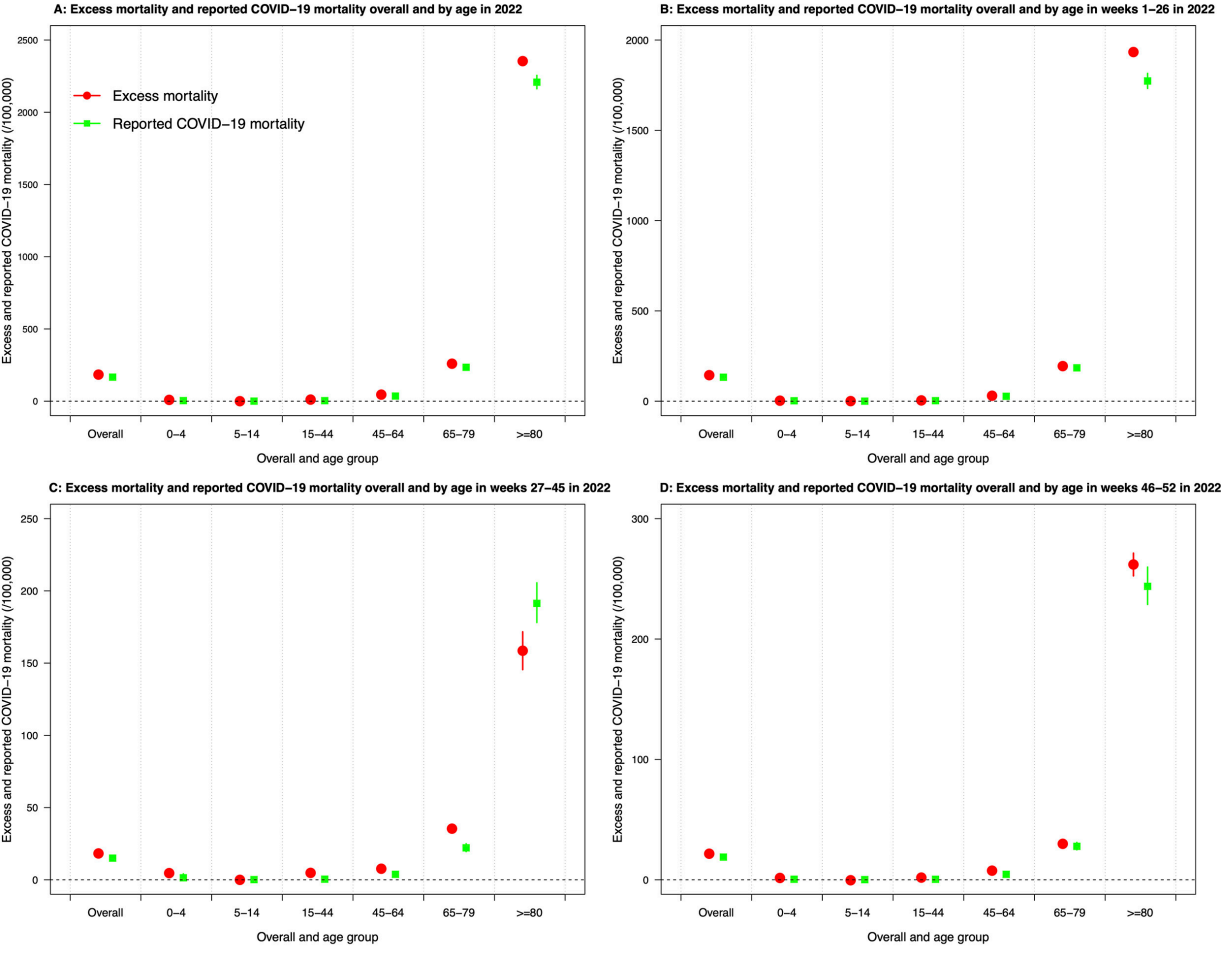
Overall and age-specific excess mortality and reported COVID-19 mortality in 2022. **Panel A.** Comparison of excess mortality and reported COVID-19 mortality overall and by age in 2022. **Panel B.** Comparison of excess mortality and reported COVID-19 mortality overall and by age in weeks 1–26 in 2022. **Panel C.** Comparison of excess mortality and reported COVID-19 mortality overall and by age in weeks 27–45 in 2022. **Panel D.** Comparison of excess mortality and reported COVID-19 mortality overall and by age in weeks 46–52 in 2022.

In total, there were 63 484 deaths (864 per 100 000 population) recorded by the death registry in 2022. With the predicted counterfactual mortality baseline of 49 966 deaths (680 per 100 000 population, 95% CI = 678, 682), the excess mortality in 2022 was estimated to be 184 per 100 000 population (95% CI = 182, 186), equating to 13 518 (95% CI = 13 403, 13 633) additional deaths and a 27% increase in all-cause mortality (**Figure 1**, Panel A, **Figure 2**, Panel A; Table S2 in the **Online Supplementary Document**). People aged 80 years or above showed the highest excess mortality risk in 2022 (2354 per 100 000 population, 95% CI = 2330, 2378) throughout the three defined epidemic periods, followed by 65–74 years (260 per 100 000 population, 95% CI = 255, 265), accounting for 68% and 22% of all excess deaths in this year, respectively (**Figure 2**; Table S2 in the **Online Supplementary Document**). Overall males (232 per 100 000 population, 95% CI = 230, 235) showed a higher excess mortality than females (143 per 100 000 population, 95% CI = 142, 145) and the sex differences were indicated in most age groups, particularly people over 80 years (n = 2897 excess deaths, 95% CI = 2858, 2935 for males *vs*. n = 1960 excess deaths, 95% CI = 1930, 1991 for females). Less than 2% of the estimated excess deaths were identified in individuals younger than 45 years (Figure S1, Panel A and Table S2 in the **Online Supplementary Document**).

Compared with 12 228 (166 per 100 000 population, 95% CI = 164, 169) individuals died with confirmed COVID-19 recorded in 2022, the slightly higher estimate of the excess mortality (184 per 100 000 population, 95% CI = 182, 186) for the same year shared similar age-, sex-, and period-specific patterns (**Figure 2**, Panel A). Older adults over 80 years of age contributed to most of the differences between excess deaths and the reported COVID-19 deaths (n/N = 567/1290 deaths increased, 44%), followed by those aged 65–79 years (n/N = 287/1290, 22%) and 45–64 years (n/N = 236/1290, 18%) (**Figure 2**, Panel A; Table S3 in the **Online Supplementary Document**). The excess mortality was also higher than the reported COVID-19 mortality for both males (232 *vs*. 212 per 100 000 population) and females (143 *vs*. 128 per 100 000 population). The sex- and age-specific analysis indicated that 63% of the overall difference between the excess and reported deaths in females was attributed to patients over 80 years of age. However, the death difference in males was contributed by the four adult groups, 15–44 (20%), 45–64 (30%), 65–79 (23%), and those over 80 years (27%) similarly (Figure S2–3 in the **Online Supplementary Document**).

**Figure 3:**
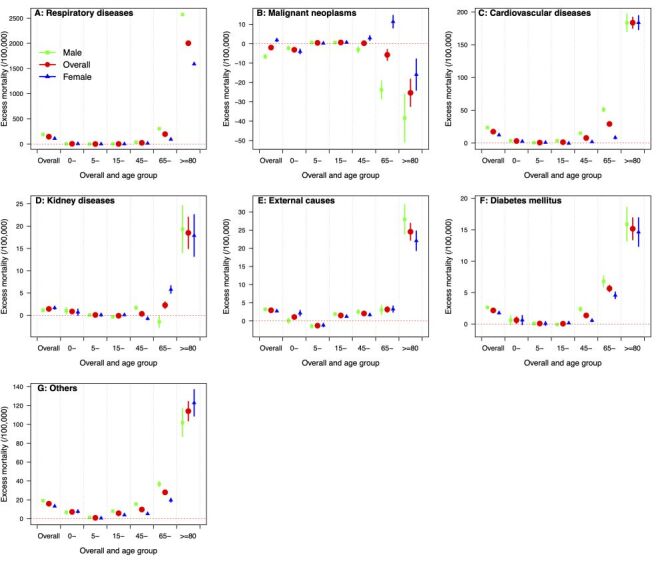
Cause-specific excess mortality by age and sex in Hong Kong in 2022. **Panel A.** Excess mortality by age and sex for respiratory diseases. **Panel B.** Malignant neoplasms. **Panel C.** Cardiovascular diseases. **Panel D.** Kidney diseases. **Panel E.** External causes. **Panel F.** Diabetes mellitus. **Panel G.** Other causes.

Among the 7 causes of death investigated in this study, excess mortality was identified in 2022 for these major causes except for malignant neoplasms (**Figure 3**; Figure S4 in the **Online Supplementary Document**). The highest excess mortality risk was estimated for respiratory diseases (145 per 100 000 population, 95% CI = 144, 146) and cardiovascular diseases (17 per 100 000 population, 95% CI = 16, 18), representing 92% and 13% increase from the estimated baseline, respectively. The additional deaths from the two causes accounted for 88% of all excess deaths estimated for 2022 (**Figure 3****;** Table S2 in the **Online Supplementary Document**). The reported mortality from malignant neoplasms (202 per 100 000 population) was slightly lower than the predicted (205 per 100 000 population, 95% CI = 204, 207), with the largest reduction in people over 80 years (reduced by 25 per 100 000 population, 95% CI = 18, 32), followed by the 65–79 years (6 per 100 000 population, 95% CI = 3, 9) (**Figure 3**, Panel B; Table S2 in the **Online Supplementary Document**). Except for malignant neoplasms, the decrease in hospitalisation was accompanied by an increase in excess mortality for most causes, with cardiovascular diseases showing the highest correlation (r = −0.77, 95% CI = −0.85, −0.62) (Figure S5–6 in the **Online Supplementary Document**).

The higher number of excess deaths over the reported COVID-19 deaths in 2022 was most prominent in period 1, with an estimated 847 more deaths (95% CI = 762, 932), accounting for about 66% (n/N = 847 / 1290) of the overall difference (**Figure 2**, Panels B–D; Table S3 in the **Online Supplementary Document**). Among these, the majority occurred in older adults over 80 years of age (n/N = 624/847, 74%) (**Figure 2**, Panel B). During period 2, however, the estimated excess deaths in people over 80 years were lower than the reported COVID-19 deaths (n = −128, 95% CI = −179, −77) although the age group 65–79 years showed the largest difference in the number of excess deaths over the reported COVID-19 deaths (n = 151, 95% CI = 120, 183) among all age groups, followed by 15–44 years (n = 116, 95% CI = 109, 123) (**Figure 2**, Panel C; Table S3 in the **Online Supplementary Document**). In period 3, people aged over 80 (n = 71, 95% CI = 34, 107) and 45–64 (n = 73, 95% CI = 60, 86) showed similar differences in the estimated excess deaths over confirmed COVID-19 deaths (**Figure 2**, Panel D; Table S3 in the **Online Supplementary Document**). The increased number of excess deaths over confirmed COVID-19 deaths was identified in all epidemic periods for both males and females (Figure S2–3 in the **Online Supplementary Document**).

## DISCUSSION

We estimated that the excess mortality in 2022 was 184 (95% CI = 182, 186) per 100 000 population, 27% higher than the baseline mortality predicted for the year based on the historical trend. The estimated excess mortality was not only identified in causes of respiratory diseases, but also other causes, such as cardiovascular diseases, accounting for 10% of the overall excess deaths in 2022, particularly among older adults at age of 80 years or above. A slightly higher number of excess deaths than the reported COVID-19 deaths was observed mainly in the older adults during the peak of the Omicron BA.2 wave in early 2022.

A close concordance was indicated in our study between the estimated number of excess deaths and the officially recorded deaths with a confirmation of COVID-19 in 2022. Globally, a report from the World Health Organization found that the excess mortality in 2020 and 2021 was 2.74 times higher than the reported COVID-19 mortality [3]. A series of excess mortality studies conducted in the US found that among the overall excess deaths estimated during the pandemic, only 65–72% were attributed to COVID-19 [16–19]. Hong Kong had very substantial laboratory testing capacity, testing approximately 3% of the population each day during March 2022 which included routine on-admission testing in hospitals [12]. Nevertheless, in our previous analysis of population mortality in Hong Kong in 2020, we found an increase in cardiovascular mortality with very few COVID-19 deaths, potentially attributable to changes in health care-seeking behaviours or disruption in care for older individuals with cardiovascular diseases [5], and this has also been reported in other locations [16–20]. In the present analysis we do not observe a substantial indirect impact of the pandemic on mortality from other causes, although some indirect impact likely occurred and is included as part of the overall excess mortality.

One notable difference between Hong Kong and other locations is that the substantial mortality impact of COVID-19 occurred in Hong Kong in 2022 whereas the highest excess mortality rates in other parts of the world were generally estimated in 2020 and early 2021 prior to the availability of COVID-19 vaccines [3,21,22]. We have reported separately that vaccine uptake in older adults in Hong Kong was low for various reasons [12,23,24], leading to a large Omicron wave in March-April 2022 [25]. We also reported that case fatality rates increased by 3-fold during the peak weeks of the Omicron wave compared to earlier and later in that epidemic [26], likely due to the extreme pressure on health care resources at that time.

Countries such as Canada and Norway, where a suppression or mitigation strategy rather than an elimination strategy was implemented to avoid overwhelming health systems in the early COVID-19 pandemic, achieved a high vaccination rate (over 70%) as in Hong Kong before the Omicron became dominant [19]. By comparing the observed overall mortality during the study period with the pre-pandemic mortality, these countries estimated a lower excess all-cause mortality than the reported COVID-19 mortality during the Omicron epidemic [19]. It could be explained that many laboratory-confirmed COVID-19 deaths, particularly in very frail older adults, were actual deaths ‘with COVID-19’ rather than ‘due to COVID-19’ who would have died anyhow if the COVID-19 pandemic did not occur. Instead, these individuals were infected with SARS-CoV-2 and were counted as COVID-19 deaths but not changing the overall mortality rate in the population [2,27]. Consequently, the death rate from causes other than COVID-19 may be reduced due to mortality displacement. This might explain the reduction we observed in deaths from malignant neoplasms in the Hong Kong population throughout 2022, as well as the relatively lower excess deaths in older adults over months after the largest omicron wave in comparison with the confirmed COVID-19 deaths.

A number of limitations exist in this study. First, we were not able to separate the direct impact from indirect impact of the COVID-19 pandemic in the estimated excess deaths. Previously Lee et al. intended to achieve the separation by including covariates representing the two types of impact in the regression model [20]. Future studies are needed to clearly define the different impact and develop valid methods to make a causal inference on the health impact caused by COVID-19. Second, the accuracy of the estimated excess mortality for some defined groups in the analysis, such as children aged 5–14 years, might be limited by the small numbers of weekly deaths. Third, unlike previous studies, often using the data before 2020 as the mortality baseline, our baseline included the data from the first two years of the pandemic, 2020 and 2021, allowing for possible structural changes in health systems and population health outcomes during the COVID-19 pandemic. The excess mortality in 2022 might be slightly underestimated or overestimated if the mortality in 2020–2021 were increased or decreased due to the pandemic although potential changes in the mortality trend in Hong Kong since the emergence of SARS-CoV-2 in January 2020 has been accounted for in the model.

## CONCLUSIONS

A substantial number of excess deaths were identified in Hong Kong during a large Omicron predominant COVID-19 epidemic, and the slightly higher number of excess deaths over the confirmed COVID-19 deaths demonstrated the direct and indirect impact from the pandemic on population mortality likely displaced during large epidemics. Our study also highlighted the importance of pandemic responses with effective pharmaceuticals in particular vaccines in reducing the overall health burden especially in the frailest population.

Additional material Online Supplementary Document

## References

[1] World Health Organization. WHO COVID-19 dashboard. 2024. Available: https://data.who.int/dashboards/covid19/cases?n=c. Accessed: 3 May 2024.

[2] Stokes AC Lundberg DJ Elo IT Hempstead K Bor J Preston SH COVID-19 and excess mortality in the United States: A county-level analysis. PLoS Med. 2021;18:e1003571. 10.1371/journal.pmed.1003571 34014945 PMC8136644

[3] Msemburi W Karlinsky A Knutson V Aleshin-Guendel S Chatterji S Wakefield J The WHO estimates of excess mortality associated with the COVID-19 pandemic. Nature. 2023;613:130–7. 10.1038/s41586-022-05522-2 36517599 PMC9812776

[4] Marijon E Karam N Jost D Perrot D Frattini B Derkenne C Out-of-hospital cardiac arrest during the COVID-19 pandemic in Paris, France: a population-based, observational study. Lancet Public Health. 2020;5:e437–43. 10.1016/S2468-2667(20)30117-1 32473113 PMC7255168

[5] Xin H Wu P Wong JY Cheung JK Lau EHY Leung GM Hospitalizations and mortality during the first year of the COVID-19 pandemic in Hong Kong, China: An observational study. Lancet Reg Health West Pac. 2022;30:100645. 36438907 10.1016/j.lanwpc.2022.100645 PMC9682934

[6] Kiss P Carcel C Hockham C Peters SAE The impact of the COVID-19 pandemic on the care and management of patients with acute cardiovascular disease: a systematic review. Eur Heart J Qual Care Clin Outcomes. 2021;7:18–27. 10.1093/ehjqcco/qcaa084 33151274 PMC7665454

[7] Woodruff BA Interpreting mortality data in humanitarian emergencies. Lancet. 2006;367:9–10. 10.1016/S0140-6736(05)67637-4 16399135

[8] Leon DA Shkolnikov VM Smeeth L Magnus P Pechholdová M Jarvis CI COVID-19: a need for real-time monitoring of weekly excess deaths. Lancet. 2020;395:e81. 10.1016/S0140-6736(20)30933-8 32333839 PMC7176374

[9] Murray CJ Lopez AD Chin B Feehan D Hill KH Estimation of potential global pandemic influenza mortality on the basis of vital registry data from the 1918-20 pandemic: a quantitative analysis. Lancet. 2006;368:2211–8. 10.1016/S0140-6736(06)69895-4 17189032

[10] Beaney T Clarke JM Jain V Golestaneh AK Lyons G Salman D Excess mortality: the gold standard in measuring the impact of COVID-19 worldwide? J R Soc Med. 2020;113:329–34. 10.1177/0141076820956802 32910871 PMC7488823

[11] Baker MG Wilson N Blakely T Elimination could be the optimal response strategy for covid-19 and other emerging pandemic diseases. BMJ. 2020;371:m4907. 10.1136/bmj.m4907 33561814

[12] Yang B Lin Y Xiong W Liu C Gao H Ho F Comparison of control and transmission of COVID-19 across epidemic waves in Hong Kong: an observational study. Lancet Reg Health West Pac. 2023;43:100969. 10.1101/2023.06.20.23291593 38076326 PMC10700518

[13] Islam N Shkolnikov VM Acosta RJ Klimkin I Kawachi I Irizarry RA Excess deaths associated with covid-19 pandemic in 2020: age and sex disaggregated time series analysis in 29 high income countries. BMJ. 2021;373:n1137. 10.1136/bmj.n1137 34011491 PMC8132017

[14] McMenamin ME Nealon J Lin Y Wong JY Cheung JK Lau EHY Vaccine effectiveness of one, two, and three doses of BNT162b2 and CoronaVac against COVID-19 in Hong Kong: a population-based observational study. Lancet Infect Dis. 2022;22:1435–43. 10.1016/S1473-3099(22)00345-0 35850128 PMC9286709

[15] Census and Statistics Department. Population Estimates. 2024. Available: https://www.censtatd.gov.hk/en/scode150.html. Accessed: 3 May 2024.

[16] Woolf SH Chapman DA Sabo RT Weinberger DM Hill L Excess Deaths From COVID-19 and Other Causes, March-April 2020. JAMA. 2020;324:510–3. 10.1001/jama.2020.11787 32609307 PMC7330820

[17] Woolf SH Chapman DA Sabo RT Weinberger DM Hill L Taylor DDH Excess Deaths From COVID-19 and Other Causes, March-July 2020. JAMA. 2020;324:1562–4. 10.1001/jama.2020.19545 33044483 PMC7576405

[18] Woolf SH Chapman DA Sabo RT Zimmerman EB Excess Deaths From COVID-19 and Other Causes in the US, March 1, 2020, to January 2, 2021. JAMA. 2021;325:1786–9. 10.1001/jama.2021.5199 33797550 PMC8019132

[19] Bilinski A Thompson K Emanuel E COVID-19 and Excess All-Cause Mortality in the US and 20 Comparison Countries, June 2021-March 2022. JAMA. 2023;329:92–4. 10.1001/jama.2022.21795 36399335 PMC9856868

[20] Lee WE Woo Park S Weinberger DM Olson D Simonsen L Grenfell BT Direct and indirect mortality impacts of the COVID-19 pandemic in the United States, March 1, 2020 to January 1, 2022. eLife. 2023;12:e77562. 10.7554/eLife.77562 36811598 PMC9946455

[21] COVID-19 Excess Mortality Collaborators Estimating excess mortality due to the COVID-19 pandemic: a systematic analysis of COVID-19-related mortality, 2020-21. Lancet. 2022;399:1513–36. 10.1016/S0140-6736(21)02796-3 35279232 PMC8912932

[22] Pallari CT Achilleos S Quattrocchi A Gabel J Critselis E Athanasiadou M Magnitude and determinants of excess total, age-specific and sex-specific all-cause mortality in 24 countries worldwide during 2020 and 2021: results on the impact of the COVID-19 pandemic from the C-MOR project. BMJ Glob Health. 2024;9:e013018. 10.1136/bmjgh-2023-013018 38637119 PMC11029481

[23] Xiao J Cheung JK Wu P Ni MY Cowling BJ Liao Q Temporal changes in factors associated with COVID-19 vaccine hesitancy and uptake among adults in Hong Kong: Serial cross-sectional surveys. Lancet Reg Health West Pac. 2022;23:100441. 10.1016/j.lanwpc.2022.100441 35359914 PMC8961079

[24] Yuan J Lam WWT Xiao J Ni MY Cowling BJ Liao Q Why Do Chinese Older Adults in Hong Kong Delay or Refuse COVID-19 Vaccination? A Qualitative Study Based on Grounded Theory. J Gerontol B Psychol Sci Soc Sci. 2023;78:736–48. 10.1093/geronb/gbac184 36416594

[25] Mefsin YM Chen D Bond HS Lin Y Cheung JK Wong JY Epidemiology of Infections with SARS-CoV-2 Omicron BA.2 Variant, Hong Kong, January-March 2022. Emerg Infect Dis. 2022;28:1856–8. 10.3201/eid2809.220613 35914518 PMC9423929

[26] Wong JY Cheung JK Lin Y Bond HS Lau EHY Ip DKM Intrinsic and Effective Severity of Coronavirus Disease 2019 Cases Infected With the Ancestral Strain and Omicron BA.2 Variant in Hong Kong. J Infect Dis. 2023;228:1231–9. 10.1093/infdis/jiad236 37368235

[27] Acosta E Global estimates of excess deaths from COVID-19. Nature. 2023;613:31–3. 10.1038/d41586-022-04138-w 36517677

